# Functional Alterations in the Olfactory Neuronal Circuit Occur before Hippocampal Plasticity Deficits in the P301S Mouse Model of Tauopathy: Implications for Early Diagnosis and Translational Research in Alzheimer’s Disease

**DOI:** 10.3390/ijms21155431

**Published:** 2020-07-30

**Authors:** Abdallah Ahnaou, Daniela Rodriguez-Manrique, Ria Biermans, Sofie Embrechts, Nikolay V. Manyakov, Wilhelmus H. Drinkenburg

**Affiliations:** Department of Neuroscience, Janssen Research & Development, a Division of Janssen Pharmaceutica NV. Turnhoutseweg 30, B-2340 Beerse, Belgium; daniela@rodriguez-manrique.com (D.R.-M.); RBIERMAN@its.jnj.com (R.B.); EMBRECH@its.jnj.com (S.E.); nmanyak1@its.jnj.com (N.V.M.); WDRINKEN@its.jnj.com (W.H.D.)

**Keywords:** Alzheimer’s disease, tau, neural oscillations, LTP, olfactory neural network, translational marker

## Abstract

Alzheimer’s disease (AD) is characterized by neuronal loss and impaired synaptic transmission, ultimately leading to cognitive deficits. Early in the disease, the olfactory track seems most sensitive to tauopathy, while most plasticity studies focused on the hippocampal circuits. Functional network connectivity (FC) and long-term potentiation (LTP), considered as the plasticity substrate of learning and memory, were longitudinally assessed in mice of the P301S model of tauopathy following the course (time and location) of progressively neurodegenerative pathology (i.e., at 3, 6, and 9 months of age) and in their wild type (WT) littermates. Using in vivo local field potential (LFP) recordings, early (at three months) dampening in the gamma oscillatory activity and impairments in the phase-amplitude theta-gamma coupling (PAC) were found in the olfactory bulb (OB) circuit of P301S mice, which were maintained through the whole course of pathology development. In contrast, LFP oscillatory activity and PAC indices were normal in the entorhinal cortex, hippocampal CA1 and CA3 nuclei. Field excitatory postsynaptic potential (fEPSP) recordings from the Shaffer collateral (SC)-CA1 hippocampal stratum pyramidal revealed a significant altered synaptic LTP response to high-frequency stimulation (HFS): at three months of age, no significant difference between genotypes was found in basal synaptic activity, while signs of a deficit in short term plasticity were revealed by alterations in the fEPSPs. At six months of age, a slight deviance was found in basal synaptic activity and significant differences were observed in the LTP response. The alterations in network oscillations at the OB level and impairments in the functioning of the SC-CA1 pyramidal synapses strongly suggest that the progression of tau pathology elicited a brain area, activity-dependent disturbance in functional synaptic transmission. These findings point to early major alterations of neuronal activity in the OB circuit prior to the disturbance of hippocampal synaptic plasticity, possibly involving tauopathy in the anomalous FC. Further research should determine whether those early deficits in the OB network oscillations and FC are possible mechanisms that potentially promote the emergence of hippocampal synaptic impairments during the progression of tauopathy.

## 1. Introduction

Neurodegenerative diseases such as Alzheimer’s disease (AD) are characterized by gradual and irrevocable damage to neural networks, leading to a progressive decline of neural plasticity and consequently to dramatic cognitive deficits. Due to the lengthy transitional state between healthy aging and sporadic AD, the potential to intervene with novel neuroprotective agents early in the course of the disease is crucial to protect against eventual reduction in quality of life. Intensive research efforts are underway to understand the processes underlying the presymptomatic stage of the disease, and to identify more sensitive biomarkers and diagnostics for early intervention strategies aimed at preventing or at least postponing the clinical onset of the disease. Several biomarkers have been identified such as amyloid beta Aβ42 and Tau levels in the cerebrospinal fluid (CSF), as well as amyloid deposition in the brain as revealed by positron emission tomography (PET) [1,2]. Tau proteins are referred to as either p-tau, which indicates hyperphosphorylated tau proteins correlated with the formation of neurofibrillary tangles in AD patients’ brains [3,4], or t-tau, which consists of different tau isomers correlated with the severity of neurodegeneration and neuronal or axonal damage [5,6]. However, these biomarkers are not widely used in clinical settings, due to either the invasiveness of the procedures and/or costs involved [1,7]. Finding a convenient, early, and robust functional index, which could be used for an accurate, inexpensive, and non-invasive diagnosis is therefore crucial.

The preferential localization of proteinopathies in the olfactory, cortex, and hippocampal structures correlates well with primary cognitive and memory disturbances [8]. In recent years there has been a rising interest in olfaction as a potential early functional biomarker in AD [9,10]. Impairments in olfactory processing are amongst the very first clinical signs that occur in almost 100% of AD cases [11], with evidence that tau (NTF) pathology in the OB may be more important than amyloid pathology underlying the early olfactory deficit in AD, clearly preceding cognitive impairments [12,13,14,15]. The close anatomical proximity of the olfactory bulb to limbic regions, together with the early occurrence of olfactory impairments in AD, underpin the hypothesis that propagation of tauopathy originates from the olfactory circuit, spreads to and to and consequently impacts the functions of limbic structures like the amygdala, entorhinal, and piriform cortices [16,17,18].

The selection of early markers of disturbance in neural network and cognitive processes is important in the identification of AD patients, as well as to identify such endpoints that might predict which individuals will ultimately progress from mild cognitive impairments (MCI) to AD. Cognitive measures assessing for example episodic memory and working memory have been widely used, while a relationship between memory and cellular mechanisms that underlies the connectivity of widespread neuronal networks and synaptic plasticity in the hippocampus is well established. The precise spike timing of activated neural networks and the temporal coordination of rhythmic oscillations play a key role in the mechanisms of synaptic plasticity and processes of cognition [19,20]. Hippocampal theta (4–8 Hz) and theta-gamma (30–100 Hz) cross-frequency coupling are key rhythms to induce synaptic plasticity, supporting learning and memory processes [19,20,21]. The link between synaptic plasticity and cognitive processes such as learning and memory is frequently studied within the hippocampus, a structure involved in diverse cognitive processes such as those related to acquisition, coding, storing, and recalling information. The phenomena of synaptic plasticity, referred to as long-term potentiation (LTP) and long-term depression (LTD) are fundamental synaptic mechanisms underlying hippocampal contributions to these processes [22,23].

Tau dysfunction has been recognized as a key pathological condition of AD. Animal models of tauopathy are generally accepted as valuable tools to identify and understand early processes underlying the initiation and spreading of tau pathology. The P301S mouse model, which expresses the mutant human microtubule-associated protein tau was developed to study mechanisms of tauopathy. Recombinant tau protein with the P301S mutation has a reduced capacity to promote microtubule assembly [24]. Tau seeding activity was observed in P301S mice at 1.5 months and increased with the age of the mice [25,26]. P301S mice additionally demonstrated a five-fold increase in extracellular monomeric tau and extracellular deposition of insoluble tau aggregates [27]. While the P301S model displayed tau filament accumulation, a decreased mitochondria number, impaired translocation of ER proteins, and axonal degeneration, it did not express Aβ plaques [28]. At three months of age, the model displayed microgliosis and small synaptic loss, whereas neuronal loss commenced from nine months and onward, likely resulting in disturbance of the sleep-wake cycle in this model [29]. At six months of age, P301S mice showed decreased sensorimotor gating in the prepulse inhibition test and exhibited deficits in spatial memory evaluated in the Morris water maze test [30]. In addition, synaptic dysfunction in the form of reduced basal synaptic activity and LTP maintenance 75 min post LTP has been observed in the hippocampal slice CA1 region [31]. However, there are still scarce in vivo studies investigating changes in synaptic plasticity across different ages in the P301S tau mouse model.

In the present study, we took advantage of the complementary information provided by local field potentials (LFPs) and LTP recordings in P301S mice to characterize how progression and spreading of tauopathy affects network functioning and plasticity indices at three, six, and nine months of age. Longitudinal changes in network oscillations and connectivity were investigated at the OB, fronto-entorhinal cortical, and hippocampal CA1 areas, while changes in in vivo LTP response were measured at the pyramidal hippocampal level.

## 2. Results

### 2.1. Network Oscillations and Connectivity

#### 2.1.1. P301S Animals Display Early Reduction in the Gamma Frequency Oscillations, Specifically in the OB Area

**Figure 1:**
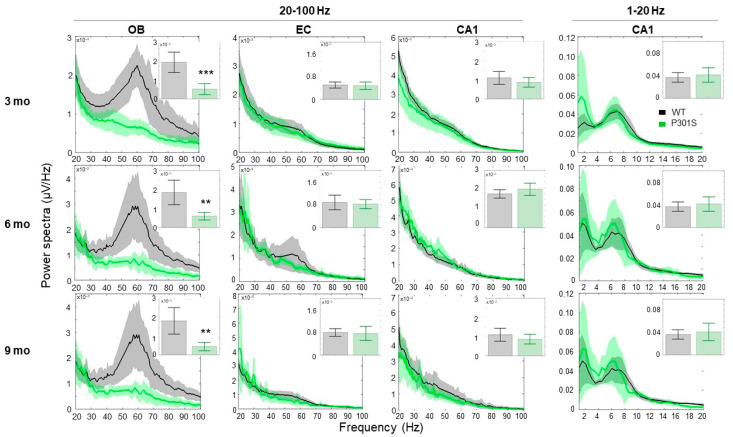
Relative power spectra for frequencies of 20–100 Hz (left panels) of LFP recorded in left OB, EC, CA1 sites, and at frequencies of 1–20 Hz (right panels) for the CA1 site for P301S (green, *n* = 8) and their WT littermates (black, *n* = 7), at recording intervals of 3, 6, and 9 months. Insets indicate total relative power with significance between group difference (two-sample *t*-test) in the 20–100 Hz and 1–20 Hz frequency range in the left and right panels, respectively. Only left hemisphere data are displayed. Data are presented as mean (across animals) values and 95% confidence intervals. Asterisks indicate the presence of a significant difference between genotypes (two-sample *t*-test), ** *p*-value < 0.01, *** *p*-value < 0.001.

At the recording month three, P301S mice displayed early impairments in the gamma oscillations range (40–80 Hz) specifically at the OB circuit (Figure 1, first raw left panel). At recording months six and nine, this reduction in the high gamma oscillations was maintained at the OB network (*p* < 0.01, two-sample *t*-test) (Figure 1, second and third left panels). No overt effect on network gamma activity was found in frontal, entorhinal cortex (EC), or the hippocampal recording sites (Figure 1, first to third middle and right panels). Quantification of the relative power in the 1–20 Hz sites did not reveal a major difference in the oscillatory rhythms between groups (Figure 1, right panels), and relative theta power did not change (Figure 1, right panels inset bars).

#### 2.1.2. P301S Animals Show Severe Early Impairments in the Theta-Gamma Phase Amplitude Coupling in the OB Region

**Figure 2:**
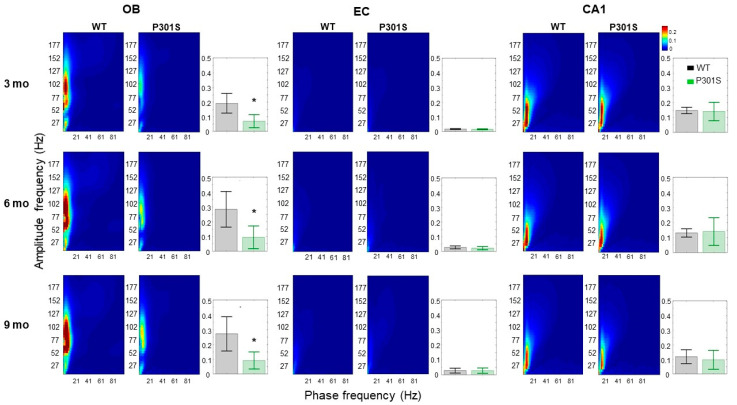
Heat maps showing the mean phase amplitude coupling (PAC) modulation index at the OB, EC, and CA1 recording electrodes for WT (left columns in each frame) and P301S (right columns in each frame) mice at recording months three, six and nine. As shown by the color scale, “hotter” colors indicate high coupling values while “colder” colors indicate low or no coupling. PAC values were computed as the average (across animals) PAC for the large window of phase frequency: 1–100 Hz, and amplitude frequency: 1–200 Hz. Bar Graphs show the mean (across animals) theta-gamma PAC (with 95% CI) at the OB, EC, and CA1 electrodes for P301S (green, *n* = 8) and their WT littermate mice (black, *n* = 7), where mean PAC index was estimated for phase 4–8 Hz and amplitude 40–100 Hz (e.g., theta-gamma coupling), and asterisks indicate the presence of a significant difference between genotypes (* *p*-value < 0.05).

The temporal interaction between superimposed network oscillations is considered as a key mechanism facilitating the communication and functional connectivity between distant brain regions required for plasticity and information processing [32,33]. Therefore, we estimated the strength of cross-frequency coupling between the phase of slow and the amplitude of fast oscillations (phase-amplitude coupling, PAC) in different recording sites. Mean PAC values at the OB regions are qualitatively shown in the form of co-modulation heat maps for WT and P301S mice. As shown in Figure 2 (left panels), WT mice demonstrated high PAC in the OB recording site, at recording months three, six, and nine. This high coupling peaks around a phase frequency of 7 Hz and amplitude frequency of around 60 Hz, in the theta-gamma range. In contrast, P301S mice demonstrate an early reduction in the strength of PAC at the OB sites at recording month three, which persists during recording sessions up to month nine.

PAC co-modulation heat maps in the hippocampal CA1 of P301S mice were indistinguishable from their WT littermates (Figure 2, bar graphs).

Overall, the data show that tau overexpression plays a role in the disturbance of normal oscillatory network activity in the OB circuit. Synaptic function and plasticity require the coordination of intra- and inter-regional activity in networks of brain structures. The alterations observed in the OB circuit may reflect early pathophysiological mechanisms, explaining a compromised processing of odor and environmental cues. Next, we hypothesized that those early network alterations could be paralleled by other forms of neurophysiological disturbance such as subtle deficits in plasticity in the hippocampus area.

### 2.2. In Vivo Electrophysiology

#### 2.2.1. Establishing Criteria, Pharmacology, and Sensitivity of the Tetanization Protocol to Modulation of the Glutamatergic NMDA Signaling

**Figure 3:**
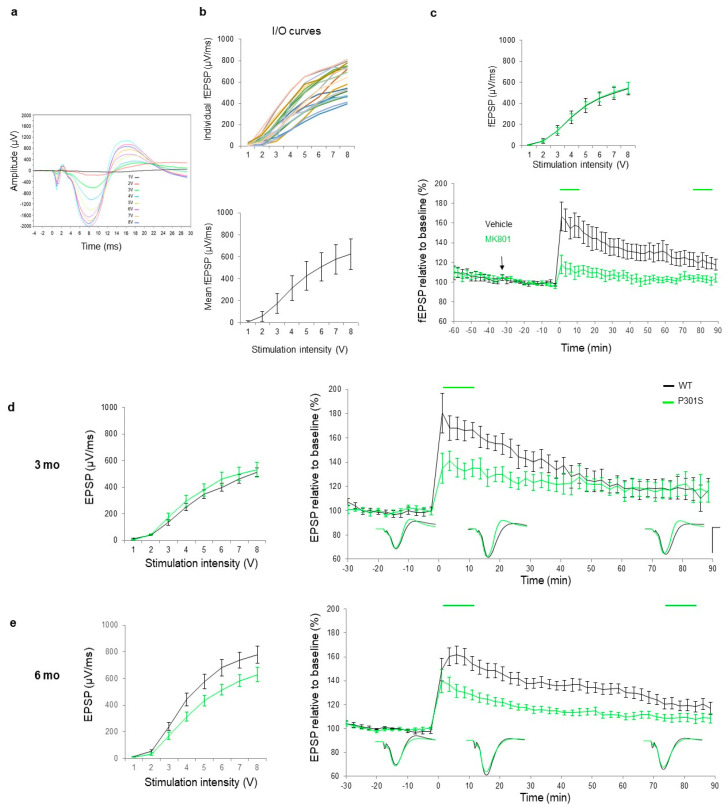
(**a**) Spaghetti curves of 21BL/6J mice who’s maximum fEPSP values at 8 V stimulation lies between 400 and 900 µV/ms. (**b**) Mean basal synaptic excitability represented in collective input/output (I/O) curves of stimulation voltage and fEPSP slopes. (**c**) Collective I/O curves of ascending stimulation voltage show an overlap in fEPSP slope response indicating no significant difference in basal synaptic excitability prior treatment with MK801. (**d**) fEPSP slope relative to the baseline is plotted across time for each group (vehicle, black (*n* = 9) and MK-801, green (0.64 mg/kg, *n* = 9). I/O curve fEPSP values were displayed for both groups). A statistical significance (indicated by the green horizontal line on top of the corresponding time interval) was found in the STP response between the two groups during the first 10 min post tetanization (*p* = 0.003) and in the LTP response at time points 70–90 min post-tetanization (*p* = 0.02). (**d**) Collective I/O curves of stimulation voltage and fEPSP slope values relative to baseline are plotted across time for 3-month-old WT (black, *n* = 8) and P301S mice (green *n* = 8) and (**e**) 6-month-old WT (black, *n* = 11) and P301S mice (green, *n* = 10), respectively. At 3 months of age, deficits were observed in the STP response, whereas at 6 months of age, deficits were found in both STP and LTP responses. A trend of impaired baseline synaptic response appeared in 6-months-of-age mice. Data are presented as means ± SEM (%). Lines above indicate statistical significance between genotypes. Of note, comparable results were observed in 9-month-old P301S mice: these data are not shown due to a low remaining sample number caused by a high mortality (e.g., sensitivity to long-term anesthesia) rate in animals at this age.

Criteria are useful measures of confidence limits to establish an appropriate cut-off basal synaptic fEPSP response at the SC-CA1 hippocampal fibers in the C57BL/6J mouse, which is a background strain for most transgenic animals of AD. With these criteria in place, fibers showing a response outside the mean margin interval could be excluded (Figure 3a,b). The two trains of 50 pulses at 200 µs pulse duration protocol showed normalized values of fEPSPs that are in line with previous reports [34,35].

The next step was to validate the sensitivity of the tetanization protocol: the main receptor involved in LTP induction at the SC-CA1 pathway is the glutamatergic NDMA signaling, therefore modulation of the NMDA receptor using MK-801, a potent non-competitive NMDAR antagonist [36,37], is expected to affect the LTP response. No significant difference was observed in I/O curves (Figure 3c, top right panel) indicating that all animals exhibited similar basal excitability. However, a significant reduction in the induction phase during the first minute and the short-term plasticity (STP) during the first 10 min post-tetanization were found in MK801-treated animals (Figure 3c, bottom right panel). A significant effect was observed at the last four time points (70–90 min) post-tetanization, suggesting that MK-801 at the dose tested had a significant effect on STP and LTP phases. These findings are thus in line with earlier observations describing long-term impairments in both spatial memory and LTP in rats [38,39].

#### 2.2.2. Plasticity Assessment in Three, Six and Nine-Month-Old P301S Mice and WT Littermates

Phenotyping the plasticity response across different ages in the P301S transgenic mice is important for modeling and understanding the changes in synaptic plasticity associated with disease progression in a tauopathy model. Accordingly, the disease pathology was sufficiently developed at six months of age to display synaptic plasticity deficiencies in P301S mice (line PS19) [31]. However, no earlier report described plasticity response at an early or late age in the current P301S mouse model of tauopathy. To assess whether pathological mechanisms were already sufficiently prevalent to cause changes in synaptic plasticity at an early age, we studied the plasticity response in P301S mice and their WT littermates at three months of age. No significant differences were observed in basal synaptic activity as revealed by I/O curve responses (Figure 3d, left panel). A significant difference was observed for the tetanization induction phase, as well as for the short-term plasticity (STP) for 10 min post-tetanization (Figure 3d, right panel). No additional differences were depicted between the groups’ fEPSP means at 70–90 min post tetanization. Therefore, the results suggest a slight emergence of difference in EPSP measures during the STP phase at three months of age.

At six months of age, the I/O curves for fEPSP values of the P301S mice were smaller than those found for the wildtype group, indicating a possibly impaired basal synaptic activity (Figure 3e, left panel), however, ANOVA analysis did not reach the significance level. A significant difference was found in the STP phase (Figure 3e, right panel), and a statistically significant difference between the two groups’ EPSP values was also observed in the LTP response between the two groups.

## 3. Discussion

In the present work, we used LFP to describe how tauopathy progression affects OB and CA1 hippocampal network oscillations and plasticity in P301S mice. We found early deficits in OB gamma oscillations associated with a decrease in peak connectivity in the OB circuit from three months onwards. This mouse strain has a deficit in STP at three months, and in LTP at six months.

### 3.1. P301S Mice Exhibited a Prominent Reduction in Gamma Oscillations at an Early Age of Three Months that Was Maintained While Getting Older

To our knowledge, this is the first study that describes the age-dependent disturbance in olfactory network oscillations in this mouse model of tauopathy. Tau deposition may occur gradually and may be dissociated from functional and behavioral alterations, while it may be correlated with the appearance of soluble phosphorylated tau as it was found in other transgenic tau models [40,41,42,43]. Thus, the disruption of neuronal function could be related to soluble tau species but not neurofibrillary aggregates. However, decreased sleep and EEG power below 8 Hz were only observed at an advanced age of 11 months in P301S mice (PS19) [29], whereas no alterations were reported on higher frequency power >20 Hz. The later study used an epidural electrode while the present study used depth electrodes to record local field potentials. We did not find alterations in frontal, entorhinal, or hippocampal recordings at 3, 6, and 9 months, however, P301S mice exhibited a prominent reduction in gamma frequency oscillations at the OB network that was maintained while getting older. As no real progress in age-related reduction was observed, deficits in the oscillations of the OB network may point to an electrophysiological endophenotype in this line of mice.

Olfactory dysfunction, a prodromal symptom of AD, is often an antecedent to classical cognitive impairments [9,10]. Gamma oscillations in the frequency range of 40–100 Hz, have extensively been studied in the OB and are the result of a negative feedback loop between excitatory mitral cells and inhibitory granule cells [44]. Experimental studies performed on a variety of species have shown a link between gamma oscillations and odor discrimination [45,46]. For example, a blockade of gamma oscillations in honeybees resulted in poor performance on discrimination tasks between similar odors [47]. In rats, gamma oscillations in the OB increased with performance difficulties in discrimination tasks [48]. Beta oscillations are associated with odor learning and discrimination, however, they rely on different brain networks [49]. Their mechanisms and functions are still largely unknown, but they are present during odor sampling [50] and their amplitude has been shown to increase with repeated exposure to odorants [51].

LFP theta band activity in the frequency range of 4–8 Hz has been observed in the OB, EC, and the hippocampus of both genotypes, while the peak frequency power was not significantly altered in P301S in comparison to WT mice. Hippocampal theta rhythm is associated with specific behavioral and cognitive processes including alertness, learning and memory, and spatial memory [19]. Coherent activity has been observed between the hippocampus and the olfactory system in task performing animals [52,53]. As the septohippocampal system is pathophysiologically affected in AD [54], impairments are therefore expected in hippocampal rhythmicity, particularly in the theta frequency range [55,56]. Previous studies showed that cognitive decline in AD is accompanied by a decrease in theta activity [55,57]. Aβ and tau have been shown to modulate cholinergic and glutamatergic activity in the septohippocampal system [58,59], and that the presence of Aβ in the medial septum and the hippocampus can dampen theta rhythm in vitro and in vivo [60,61], which is associated with cognitive impairments. Here, we did not find significant power differences in local slow and fast theta band activity in CA1 between genotypes at the ages tested, which is in line with recent work in the model.

### 3.2. P301S Mice Exhibited Early Connectivity Deficits at the OB Circuit

Synaptic function and plasticity require the coordination of intra- and inter-regional activity in networks of brain structures. Theta-gamma PAC has been widely investigated because of its key roles in learning and memory processes. In electrophysiological studies, gamma frequency stimulation bursts repeated at theta frequency effectively induces long-term potentiation, a type of synaptic plasticity in the hippocampal CA1 area [20,62]. The strength of the PAC index has been shown to correlate with cognitive performance [32,33]. Tau and Aβ pathologies can account for several dysfunctional neuronal networks associated with cognitive deficits symptoms. Impaired theta-gamma PAC has been demonstrated prior to Aβ accumulation in an amyloid mouse AD model [63] and in a tau seeding model [64,65], and has been suggested to be an early functional biomarker of AD [66]. In addition, AD patients exhibit impairments in olfaction information processing that can be observed in prodromal stages of the disease, before manifestations of cognitive symptoms [67,68,69]. Similarly, abnormal cross-frequency coupling has been demonstrated in AD models and patients [64,65,70,71,72,73]. In the present study, P301S mice exhibited large decreases in gamma oscillations in the OB circuit, which occurred in the absence of significant changes in the power of the theta oscillations, resulting in significant alterations in the theta-gamma PAC mainly in the OB circuit. The early network alterations observed in the OB circuit are consistent with early tau pathology in the OB, and a disturbance in the oscillations and connectivity of the OB network may reflect an early pathophysiological mechanism and a putative endophenotype explaining a compromised odor and a cognitive processing of environmental cues.

### 3.3. P301S Mice Exhibited Deficits in LTP Response at Six Months of Age

Phenotyping the plasticity response in P301S and WT mice at three and six months of age showed significant differences in the early phase of the LTP response. Comparable results were observed in nine-month-old P301S mice; however, these data are not shown due to a low remaining sample number following a high mortality rate in animals at this age.

At three months of age, slight differences in synaptic plasticity between P301S and WT mice were observed in their EPSP values. In the present study, recording electrodes were placed in stratum pyramidal to record fEPSPs and population spike amplitude (PSA). fEPSPs are useful extracellular measures of synaptic input to the CA1 area, whereas the PSA reflects the summed firing of a large number of pyramidal neurons in response to excitatory drive. Considering the different distribution of NMDA and AMPA receptors over synaptic sites in the CA1 hippocampal region and their role in the synaptic response [74] a difference in their quantal behavior might be expected at early disease progression stages. LTP is associated with an increase in response sensitivity caused by an increment in AMPA receptors, which are enriched in the postsynaptic membrane on dendritic spines. Tau in vivo binds to microtubules and actin simultaneously [75]. The protein plays a key role in coordinating microtubule and actin networks for several neuronal functions. Changes in cytoskeletal actin could mediate toxicity in tauopathies [76], since tau has been shown to interact to stabilize actin. Tau seeding activity at one and a half months and hyperphosphorylated tau depositions at one month of age have been reported in P301S mice [25,26]. Therefore, abnormal actin polymerization at the dendritic spines, constraining of PD growth and receptor recruitment could be a result of early tau seeding and hyperphosphorylated tau activity in the P301S model. This effect could manifest itself in a reduced recruitment of AMPA receptor activation resulting in decreased EPSP values post tetanization. Further investigation into cellular mechanisms using voltage clamp methods may help to identify the specific factors involved. 

### 3.4. At Six Months of Age, a Clear Trend of Impairments Was Observed in EPSP I/O Values and a Significant Decay Was Observed in the LTP Response in P301S Mice

Yoshiyama et al. [31] reported a significantly impaired basal synaptic activity in P301S (line PS19) mice compared to non-transgenic littermates. I/O curve EPSP values were lower at the same stimulation voltages, and maximum EPSP values of transgenic mice were reduced compared to wildtype. Our results showed that LTP fEPSP responses were significantly higher for STP, whereas differences between genotypes disappeared at later recording time points. The slight differences with [31] could be due to the difference in protocols used, as a paired pulse facilitation and theta burst stimulation were used, whereas the present work used an HFS tetanization protocol. Here, a reduction in basal synaptic transmission was not observed early at three months, while only STP was impacted after the tetanization. Other electrophysiological studies in transgenic tau models have reported somewhat contrasting results such as decreased basal synaptic activity in the absence of disturbed LTP response [77], or in association with deficits in LTP response [31] or impaired LTP only [78,79]. Inconsistencies in those studies are likely caused by the variable expression level of the transgene and tetanization protocols. 

### 3.5. Translational Perspective

Clinical trials for disease-modifying drugs in AD have failed thus far due to a lack of efficacy on cognitive, symptomatic endpoints, while testing in already advanced pathophysiological stages of AD patients using late phase biomarkers. Cognitive deficits, which are the main clinical endpoint indices are typically diagnosed late in the disease progression at a time when irreversible processes have already occurred. Sensory processes such as smelling, hearing, and vision are naturally declining to some extent in aged humans but have been shown to be prodromal of dementia [9,10]. Worsening of olfaction impairments are subsequently predictive of MCI developments and may predict the progression to AD [80,81,82,83]. Several clinical studies have demonstrated early tau pathology in the anterior OB circuit, confirming that dysfunctional olfaction may precede cognitive loss [67,69,84], which indicates a concrete potential for the use of olfaction circuitry function in diagnosis and translational research.

The abolishment in gamma network oscillations observed in the present tau mouse model may result from neurodegeneration or neurodevelopmental deficits. Therefore, the translational value of the observation is highly dependent on this and the most straightforward way to assess this is to conduct an olfactory discrimination study in three-month-old mice. Deficits in gamma oscillations may result from the excessive p-tau expression in the OB as it was observed in P301S mice (PS19 line), where decreased firing rates of the mitral olfactory cells was observed in two-month-old P301S mice [85]. An immunohistochemical study carried out in the P301S tau mouse model used in the present study, showed a progressive tau pathology in the olfactory bulb and the piriform cortex starting at one month of age and noticeable neuronal loss in the piriform cortex from the age of three months [86]. In addition, olfactory sensitivity for social or non-social odors was significantly impaired at three months of age [86]. Moreover, decoding stimulus features during odor learning and discrimination has been related to the strength of PAC in the OB area involved in early sensory processing [87]. Thus, early deficits in gamma network oscillations and FC described in the present study may underly the olfactory dysfunction including impaired odor discrimination observed in three-month-old P301S tau [86].

In addition, the induction of gamma oscillations using visual flicker or auditory tone stimulation caused a generalized reduction in amyloid plaques throughout the neocortex of AD mouse models and a reduction in phosphorylated tau in the P301S mouse model of tauopathy [88,89]. The marked deficits in gamma oscillatory rhythm found early in the OB of P301S mice prompt the question of whether entrainment of neural activity at gamma frequency rhythm would have protective functional effects. Future experiments will use gamma entraining oscillations and spiking at 60 Hz to explore the functional connection between early deficits in OB gamma rhythms and late deficits in hippocampal CA1 synaptic plasticity.

Overall, the early and most robust spontaneous deficits in the OB circuit, under physiological conditions in conscious and unrestrained animals, together with later progressive deficits at the hippocampal level support the potential of the OB circuit as a potential early neurophysiological index of dysfunctional olfaction circuitry and function associated with tauopathy.

## 4. Materials and Methods

### 4.1. Animals

All experimental procedures were conducted in accordance with the guidelines of the Association for Assessment and Accreditation of Laboratory Animal Care International (AALAC) and with the European Communities Council Directive of 24th November 1986 (86/609/EEC) and were approved by the local ethical committee. Experiments were performed on male transgenic mice expressing human P301S Tau protein created at the lab of Michel Goedert (MRC, Cambridge, UK), backcrossed to C57BL/6J at Jackson Labs. Animals were group-housed with their littermates in ventilated cages kept under controlled conditions with a 12 h/12 h light/dark cycle (lights on at 7 p.m.) and had ad libitum access to food and water.

### 4.2. In Vivo Local Field Potential (LFP) Procedures

#### 4.2.1. Surgery

**Figure 4:**
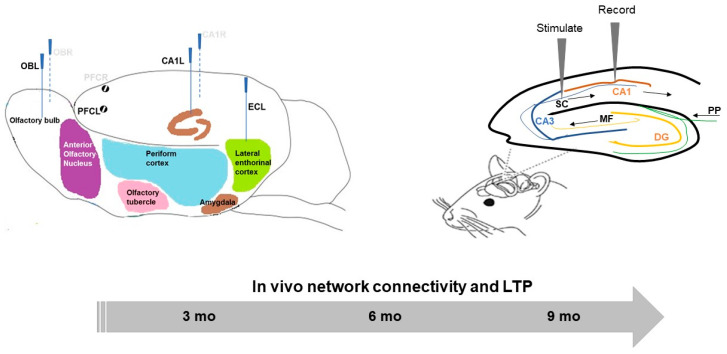
Scheme showing on the left panel a placement of LFP recording electrodes (olfactory bulb: OB, prefrontal cortex: PFC, entorhinal cortex: EC, hippocampal CA1, left: L) in conscious, freely moving mice, and on the right panel a placement of the stimulation-recording path (Schaffer collateral-CA1 synapses) in anesthetized mice (DG: dentate gyrus, PP: perforant pathway, MF: mossy fiber, CA: hippocampal cornu ammonis).

Surgery was carried out in transgenic and wildtype (WT) C57BL/6J mice weighing between 20 and 28 g at the time of electrode implantation. Animals were anesthetized with isoflurane and were mounted in a stereotaxic frame equipped with a heating pad to maintain their core body temperature at 38 °C. Animals were then stereotaxically equipped with seven stainless steel recording electrodes in the olfactory bulbs (OB) (AP: 4 mm from Bregma, ML: ±1.2 mm, DV: −2 mm), frontal cortex (AP: +2 mm from Bregma, ML: ±1.4 mm) the lateral entorhinal cortex (EC)(AP: −2.9 mm from Bregma, ML: −3.7 mm, DV: −1.7 mm) and the hippocampus CA1 (AP: −1.7 mm from Bregma, ML: ±1.5 mm, DV: −1.7 mm)(Figure 4, left). A reference electrode was placed above the midline of the cerebellum. Electrodes were connected to a pin with a small insert (Future Electronics: 0672-2-15-15-30-27-10-0) (Track pins; Dataflex: TRP-1558-0000) and were inserted into a 10-hole connector, which was carefully fixed to the skull with dental cement.

#### 4.2.2. Experimental Design, Recording, and Analysis

Following a one-week recovery period and adaptation to recording conditions, LFP was recorded once a month at three, six, and nine months of age. Recordings were performed in the animal’s home cages during the dark phase of the circadian cycle, as described elsewhere [64,65]. Motor activity was measured by a pair of passive infrared (PIR) detectors located above every recording cage. Continuous LFP recordings were acquired for 20 h with an input range of +/− 500 mV through a Biosemi ActiveTwo system (Biosemi, Amsterdam, The Netherlands). Signals were amplified, analog band-pass filtered between 1 and 256 Hz, and then digitized to a 512 Hz sampling rate with 24-bit resolution.

#### 4.2.3. LFP Spectra

Analysis was performed using a MATLAB toolbox described earlier [64,65]. Briefly, spectral power density was calculated in 2 s sliding windows using a Welch’s method with Hanning window, and the power spectra were expressed as relative power for each frequency over 1–256 Hz. Average across recording time relative power in each frequency bin of each location was averaged across animals for WT and P301S mice separately to visualize the grand averaged relative spectra. For the sake of clarity in presenting spectral data, graphs only showed the frequency range between 1–20 Hz and from 20–100 Hz. To investigate the significance of between genotype differences, mean relative spectral power between the study groups for a particular frequency band was analyzed. 

#### 4.2.4. Phase-Amplitude Cross-Frequency Coupling

To estimate whether high-frequency LFP amplitudes are modulated by low-frequency phase variations for the same electrode site signals, phase-amplitude coupling (PAC) was calculated using the algorithm based on modulation index (MI) [64,65]. MI is estimated as a mean (along time *t*) absolute value of the signal z(*t*) = A_H_(*t*)∙exp(i∙φ_L_(*t*)), $i=\sqrt{-1},$ using instantaneous phase φ_L_(*t*) derived via Hilbert transform from narrow band-pass filtered signal around low-frequency f_L_, and instantaneous amplitude envelope A_H_(*t*) derived via Hilbert transform from narrow band-pass filtered signal around high-frequency f_H_. For PAC estimation, f_L_ was varied in the interval 1–100 Hz with a step of 2 Hz, and all f_H_ taken from interval 10–200 Hz with a step of 5 Hz were considered.

### 4.3. In-Vivo Electrophysiology Procedure

#### 4.3.1. Surgery

An incision was made along the midline of the head to insert a bipolar stimulating electrode of tungsten wire (0.5 MΩ impendence and 1–2 µm tip diameter, World Precision Instruments) into the Schaffer collateral (SC) and a monopolar recording electrode of Teflon/-coated tungsten wire (75 µm outer diameter) into the stratum pyramidal layer of the CA1 (Figure 4, right panel). The coordinates for the SC are AP: −2, 0; ML: −2, 0; DV: ~1.2 and for the stratum pyramidal layer are AP: −1, 7; ML: −1, 5; DV: ~1.1 from dura (Figure 4).

The first part of the LTP study carried out in WT C57BL/6 mice evaluated optimal protocol conditions for the stimulation and recording of the LTP response. Stimulation of SC fibers at different voltages revealed cluster responses in the input/output curves of the stratum pyramidal layer of the CA1 with cut-off responses observed between 400 and 900 µV/ms for EPSP values at the maximum stimulation of 8 V. The HFS protocol of 2 trains of 50 pulses each at 200 µs pulse duration was used. Complications with anesthesia have been described in the electrophysiology literature in mice. Inconsistent results were observed with higher doses of urethane compared to those typically used in rats [35,90,91]. Pilot studies using urethane proved unsuccessful since mice typically needed incremented doses, which resulted in hypersalivation and lethality. Sodium pentobarbital was thus implemented as an anesthetic to align with previous reports [31,34,92]. 

#### 4.3.2. Basal Synaptic Activity and Inclusion and Exclusion Criteria

Single square pulses (200 µs, 3000 mV) were delivered using a constant current isolator unit (Multichannel System MC SRG4002), while descending the recording and stimulating electrodes (at 0.2 mm/min), to confirm their location in the brain. Labview homemade oscilloscope software was used to visualize the evoked field excitatory postsynaptic potentials (fEPSPs) from the stratum pyramidal in the CA1 area in response to stimulation of the ipsilateral SC pathway. Basal excitability was evaluated by generating an input/output (I/O) curve that measured the slope of the fEPSP responses across a range of stimulation voltages.

fEPSP responses need to meet inclusion and exclusion criteria prior to engaging in an I/O curve: the latency to peak negative deflection of fEPSPs is within 6–10 ms, the maximum amplitude between 1500 and 2500 µV and its maximum slope must lie between 400 and 900 µV/ms at 200 µs stimulus duration (Figure 3). Once the response met pre-set criteria, a functional I/O curve is generated. Stimulation at intensities ranging from 1 to 8 V in steps of 1 V at 0.033 Hz frequency and 200 µs duration were delivered. The curve was drawn using the mean of the three responses at each time point (Figure 3a,b). The stimulus that evoked an fEPSP slope of 50% of the maximum response was selected as a test stimulus for the LTP induction procedure. All I/O curves followed a sigmoid pattern and the calculated test stimulus fit between 3300 and 4700 mV for all experiments. 

#### 4.3.3. LTP Induction

fEPSPs were recorded using a Biosemi Active Two amplifier (Differential amplifier, Netherlands) at a sample rate of 3 kHz. The high-frequency stimulation (HFS) protocol that was used to induce LTP response, consisted of two trains of 50 pulses at 200 µs pulse duration with an inter-train interval of 30 s and 100 Hz frequency [34,35]. For each time point measured during the experiment, five records of evoked responses at a frequency of 0.033 Hz and 200 µs duration were averaged. The duration of the experiment is 30–60 min baseline followed by 90 min after tetanization. The last 30 min of the baseline recording (six time points) were averaged and used as a control for LTP induction. 

### 4.4. Genotype and Histological Confirmation of Recording Sites

At the end of the experiments, tails of the P301S mice were snipped and collected to confirm the genotype of animals using the PCR with flanking primers. To confirm immunohistochemically the stimulation and recording sites, electrical lesioning was used to determine whether the coordinates were on the SC-CA1 pathway. Animals in which the results of the two tests varied, were disqualified. 

### 4.5. Pharmacological Validation

MK-801 (Sigma Aldrich) was used as a pharmacological reference of reliable LTP induction. Vehicle or MK-801 (0.64 mg/kg) was administered intraperitoneally 30 min prior to tetanization in a volume of 1 mL/100 g body weight.

### 4.6. Data Analysis

Result for described LFP metrics and for groups of P301S and WT animals are presented as mean values with 95% confidence intervals (CI). The between-group difference in means was assessed using a two-sample *t*-test and any significance is indicated by asterisks on box plots (* *p*-value < 0.05, ** *p*-value < 0.01, *** *p*-value < 0.001). All LTP data were expressed as a percentage of change from baseline and were presented as means ± SEM %. The slope of the fEPSP was calculated from the least square linear fit performed on the 80% interval between the artifact end and the negative peak. fEPSP slopes were obtained every 2.5 min as an average of five responses at 0.033 Hz. Repeated measures analysis of variance (ANOVA) followed by a post-hoc test (Dunnett’s test) were used to correct for multiple comparisons. The difference in means between groups was considered significant if the *p*-value was below 0.05.

## 5. Conclusions

Overall, our study demonstrates over long-term recording conditions a reduction in gamma oscillations associated with impairments in the strength of PAC at the OB network that was not age dependent. The selective development of electrophysiological changes in P301S mice highlights the specific vulnerability of neuronal networks to tau pathology and associated disruptions in neuronal communication. Further research should determine whether those early deficits in OB network oscillations and FC are possible mechanisms that potentially promote the emergence of hippocampal synaptic impairments in the progression of tauopathy.
